# Deciphering the Virus Signal Within the Marine Dissolved Organic Matter Pool

**DOI:** 10.3389/fmicb.2022.863686

**Published:** 2022-05-27

**Authors:** Mara E. Heinrichs, Benedikt Heyerhoff, Berin S. Arslan-Gatz, Michael Seidel, Jutta Niggemann, Bert Engelen

**Affiliations:** ^1^Benthic Microbiology Group, Institute for Chemistry and Biology of the Marine Environment, Carl von Ossietzky University, Oldenburg, Germany; ^2^Research Group for Marine Geochemistry (ICBM-MPI Bridging Group), Institute for Chemistry and Biology of the Marine Environment, Carl von Ossietzky University, Oldenburg, Germany

**Keywords:** FT-ICR-MS, vDOM, virocell, ultrahigh-resolution mass spectrometry, bacteriophages, Roseobacter, mitomycin c, prophage induction

## Abstract

Viruses are ubiquitously distributed in the marine environment, influencing microbial population dynamics and biogeochemical cycles on a large scale. Due to their small size, they fall into the oceanographic size-class definition of dissolved organic matter (DOM; <0.7 μm). The purpose of our study was to investigate if there is a detectable imprint of virus particles in natural DOM following standard sample preparation and molecular analysis routines using ultrahigh-resolution mass spectrometry (FT-ICR-MS). Therefore, we tested if a molecular signature deriving from virus particles can be detected in the DOM fingerprint of a bacterial culture upon prophage induction and of seawater containing the natural microbial community. Interestingly, the virus-mediated lysate of the infected bacterial culture differed from the cell material of a physically disrupted control culture in its molecular composition. Overall, a small subset of DOM compounds correlated significantly with virus abundances in the bacterial culture setup, accounting for <1% of the detected molecular formulae and <2% of the total signal intensity of the DOM dataset. These were phosphorus- and nitrogen-containing compounds and they were partially also detected in DOM samples from other studies that included high virus abundances. While some of these formulae matched with typical biomolecules that are constituents of viruses, others matched with bacterial cell wall components. Thus, the identified DOM molecular formulae were probably not solely derived from virus particles but were partially also derived from processes such as the virus-mediated bacterial cell lysis. Our results indicate that a virus-derived DOM signature is part of the natural DOM and barely detectable within the analytical window of ultrahigh-resolution mass spectrometry when a high natural background is present.

## Introduction

Marine dissolved organic matter (DOM) is one of the largest actively exchanging organic carbon reservoirs on Earth, holding approximately as much carbon (C) as the atmospheric carbon dioxide pool (Hedges, 1992; Hansell et al., 2009). DOM is per operational definition separated from particulate organic matter by size filtration (Nagata, 2008). Accordingly, any organic matter that passes through filters of a nominal pore-size of 0.7 μm is defined as ‘dissolved.’ Besides truly dissolved molecular species, the filtrate encompasses any other organic matter, for example colloidal material and particles such as small prokaryotic cells and viruses (Isao et al., 1990; Ogawa and Tanoue, 2003; Breitbart et al., 2018). Besides C, DOM contains nitrogen (N), phosphorus (P), sulfur (S), and can be associated with iron or other elements that are essential to promote growth of marine organisms (Dittmar and Stubbins, 2014). Due to its enormous pool size and bioreactivity, DOM plays an important role in global biogeochemical cycles and in many ecological processes (Azam et al., 1983; Hansell, 2013; Repeta, 2015). While the main source of marine DOM is photosynthetic primary production in the euphotic zone, particularly labile DOM compounds are transformed by heterotrophic prokaryotes and enter the marine food web via the microbial loop (Azam et al., 1983; Buchan et al., 2014). A significant proportion of these prokaryotes is infected by viruses at any given moment (Fuhrman, 1999; Suttle, 2005; Zimmerman et al., 2020).

Viruses are the most abundant biological entities in the ocean (Suttle, 2005) and have been found in all investigated habitats where cellular life exists, from the uppermost layer of the sea-surface far into deep subsurface sediments (Suttle, 2005; Engelhardt et al., 2011; Lara et al., 2017; Rahlff, 2019). Here, we use the term viruses for bacteriophages. Upon infection, lytic viruses immediately force their hosts to produce and release virus progeny, while lysogenic viruses pursue a different replication strategy. They integrate into the prokaryotic genome as prophages and simultaneously propagate along with their host during cell division. When lysogenic cells encounter a DNA damaging agent, the prophages can be activated (or ‘induced’) and enter the lytic production cycle (Paul, 2008). By doing so, lytic viruses daily remove up to 40% of the prokaryotic standing stock. This so called ‘viral shunt’ causes the release of large amounts of cell material in form of particulate and DOM and high numbers of newly produced virus particles into the environment (Wilhelm and Suttle, 1999; Suttle, 2007). In recent years, the composition and turnover of virus-mediated lysates of different marine organisms was increasingly investigated, for example in single cyanobacterial strains (Ankrah et al., 2014; Ma et al., 2018; Zhao et al., 2019; Zheng et al., 2021), benthic bacterial communities (Heinrichs et al., 2020) and eukaryotic phytoplankton (Kuhlisch et al., 2021). This lysate was coined virus-induced DOM (vDOM) by Zhao et al. (2019) and is composed of cell material in particulate and dissolved form as well as virus particles. The vDOM signature was found to be compositionally distinct and particularly complex compared to the ambient DOM pool and remarkably similar on bulk molecular levels independent of the source organism (Ma et al., 2018; Zhao et al., 2019; Heinrichs et al., 2020). So far, it is not known which fraction of this vDOM fingerprint stems from the virus particles themselves.

As virus particles basically represent genomes enclosed by a protein coat, they are primarily composed of nucleic and amino acids (Jover et al., 2014). Thus, viruses are enriched in N- and P-bearing compounds compared to an average bacterial cell and are assumed to significantly contribute to N cycling and the pool of dissolved organic P in the marine environment (Wilhelm and Suttle, 1999; Shelford et al., 2012; Jover et al., 2014). Due to the vast abundance and infectivity of viruses, the compounds and elements bound in virus particles are an integral part of biogeochemical cycles in the ocean. These components, which are released into the marine DOM pool upon virus-mediated cell lysis, are recycled within the marine food web and can be transported throughout the oceans (Gonzalez and Suttle, 1993; Noble and Fuhrman, 1999; Weinbauer, 2004; Dell’Anno et al., 2015). Roughly 0.2 Pg of C are stored in marine virus particles as a direct consequence of their high abundances in the ocean (Suttle, 2013). In extreme old and oligotrophic sediments with high virus-to-cell ratios (VCRs), the carbon bound to viruses equals or even exceeds the amount of prokaryotic biomass (Engelhardt et al., 2014). While the total amount of carbon bound to viruses is quantitatively relatively low compared to the global inventory of marine DOM (660 Pg of C; Hansell et al., 2009), viruses play an important role in labile DOM cycling (Jover et al., 2014; Dell’Anno et al., 2015). Although the oceanographic size class definition of DOM clearly includes virus particles, it is unknown if a virus-derived DOM signature can be detected by state-of-the-art DOM analytics.

Until today, the molecular characterization of DOM remains an analytical challenge due to its enormous chemical diversity (Zark et al., 2017; Hawkes et al., 2018). While it is still impossible to resolve the structural composition of DOM on a broad scale, ultrahigh-resolution Fourier-transform ion cyclotron resonance mass spectrometry (FT-ICR-MS) coupled to electrospray ionization (ESI) is one of the most advanced techniques for the analysis of marine DOM so far (Riedel and Dittmar, 2014). Due to the high resolution, thousands of individual masses can be detected, and molecular formulae can be assigned to the masses within a single sample, allowing for an analysis on a bulk molecular formulae level (Stenson et al., 2003). However, some compounds are not retained by the commonly applied solid-phase extraction (SPE) via commercially available PPL resins (Dittmar et al., 2008) and might not be targeted by mass spectrometry. The polymer of the PPL cartridge, that is used for concentration and desalting of DOM samples primarily recovers uncharged, slightly polar compounds in a size range of 100 to 1,000 Da. Very small and polar compounds, such as amino acids, sugars or N-containing compounds are poorly retained (Hertkorn et al., 2013; Hawkes et al., 2016; Raeke et al., 2016; Johnson et al., 2017). These compounds presumably represent the main constituents of viruses. Concerning FT-ICR-MS analyses, our standard ESI and instrument settings are optimized to detect singly charged molecules in a mass range <2,000 Da, not allowing the ionization and detection of intact viruses, but rather individual molecular fragments deriving from virus particles (Fuerstenau et al., 2001). Despite their sheer abundance, viruses may not be quantitatively relevant owing to their small molecular weight as shown in previous experiments where viruses did not contribute significantly to DOC and DON that was released during incubation (Lønborg et al., 2013; Zhao et al., 2019). Thus, it is not clear if a virus-derived DOM signature can be deciphered from the natural marine DOM background within the analytical window of routine DOM analyses via FT-ICR-MS following filtration and SPE. Considering their ubiquitous distribution within the marine environment, it is of high interest to assess a putative virus signal within the DOM pool to identify their contribution to the global cycling of carbon and other elements.

In our study, we used the lysogenic bacterium *Rhodovulum sulfidophilum* as a model organism, as it belongs to the ecologically important and abundant family of *Rhodobacteraceae.* Members of this family are widely distributed within the marine environment and play key roles in biogeochemical cycling (Buchan et al., 2005; Moran et al., 2010; Simon et al., 2017). The organism is representative for pelagic, but also benthic habitats and contains a prophage. In this study, we tested if a molecular signature deriving from virus particles leaves an imprint on the DOM pool using routine DOM preparation and analysis procedures. More specifically, we were interested if a virus signal can be extracted from (i) DOM of a lysed bacterial culture (vDOM), (ii) the natural DOM background of seawater and (iii) if so, in which DOM size class it can be detected. To address this, we induced the prophages of *R*. *sulfidophilum* using the DNA-damaging antibiotic mitomycin C (Paul, 2008) to produce a virus-induced cell lysate. Moreover, we sampled North Sea water (NSW) containing a natural microbial community. To increase the virus signal compared to the natural background, one experimental setup of the NSW was spiked with vDOM of *R. sulfidophilum*. The cell lysate and NSW were then subjected to serial filtration steps with filter sizes following oceanographic size class definitions. Those were the particle-associated bacterial fraction (pore size >3 μm), the DOM fraction (<0.7 μm) and the free-living bacterial fraction (>0.22 μm) as well as a bacteria-free virus fraction (<0.22 μm) and the virus-free permeate (<0.02 μm). The vDOM and DOM samples of all size classes were solid-phase extracted (Dittmar et al., 2008) and their molecular composition analyzed using FT-ICR-MS. After defining a DOM signature of the viruses, we checked all filter fractions for the recovery of defined molecular formulae attributed to this signature and compared the virus signature derived from the vDOM and marine DOM dataset.

## Materials and Methods

### Preparations

Before usage, all materials were acid-washed (ultrapure water, pH 2, HCl 25%) and carefully rinsed with ultrapure water. Glassware was additionally combusted for 3 h at 500°C to avoid any contamination with organic carbon compounds. All reagents used were at least analytical grade. Cleanliness of the used material and media was monitored prior to and during the experiment by running procedural dissolved organic carbon (DOC) blanks.

### Cultivation of the Bacterial Strain

The lysogenic strain *R. sulfidophilum* P122A was used as model organism to test if a virus signal can be detected in the vDOM fingerprint of the bacterial culture upon prophage induction. The strain was isolated in 2002 from a water depth of 4,813 and 42 m below the seafloor from sediments, which were collected in the framework of the Ocean Drilling Program at site 1,231 during leg 201 (Batzke et al., 2007). A detailed description of the sampling site, isolation procedure and phylogeny are given in D’Hondt et al. (2004) and Batzke et al. (2007). For the experiment, *R. sulfidophilum* cells were inoculated from a glycerol stock and grown in triplicates in artificial seawater medium (ASW). The ASW was prepared as described by Zech et al. (2009), with the sole modification of excluding EDTA from the trace element solution. It was further supplemented with 10 mM of glucose as carbon source and 1 mM of a vitamin solution (Balch et al., 1979). Before starting the experiment, the purity of the bacterial culture was confirmed by 16S rRNA sequencing (see Supplementary Material). Therefore, single colonies from a fresh Agar plate of an actively growing culture of *R. sulfidophilum* were picked and placed in an ultrasonic bath for 15 min at 20°C to lyse the cells. 16S rRNA gene fragments were amplified by polymerase chain reaction using Green Taq polymerase (6 U, Biotechrabbit) and the primers 27f (5′-AGA GTT TGA TCC TGG CTC AG-3′) and 1492r (5′-GGT TAC CTT GTT ACG ACT T-3′) (Weisburg et al., 1991) with following cycler settings: 4 min of activation at 94°C, 32 cycles of denaturation for 30 s at 4°C, annealing for 45 s at 57°C and 1 min of elongation at 72°C, finishing with a final elongation step of 10 min at 72°C. PCR products were purified using the QIAquick PCR Purification Kit (QIAGEN) and sequenced by Eurofins.

*R. sulfidophilum* P122A contains the prophage RS1 that was isolated previously via mitomycin C induction and fully genome sequenced (Engelhardt et al., 2011). Its genome represents a 40,231 bp contiguous sequence of linear double-stranded DNA with a G + C content of 32%, including 57 protein encoding genes which were annotated using Rapid Annotation using Subsystem Technology (Aziz et al., 2008; Overbeek et al., 2014; Brettin et al., 2015). As seen from transmission electron microscope imaging, *Rhodovulum* phage RS1 is a bacteriophage with a tail of 100 nm length and a capsid of 46 nm in diameter, belonging to the *Siphoviridae* family (Engelhardt et al., 2011). The complete genome sequence of *Rhodovulum* phage RS1 is available in GenBank under accession number JF974307.1.

Due to the time-consuming filtration procedure and limited material, the three biological replicates were prepared in subsequent order. *R. sulfidophilum was cultivated in Schott Duran borosilicate glass bottles* (*GL 45, Glass type 1, red PBT lid, wadded with PTFE*) *and Duran Super Duty Erlenmeyer flasks.* Cells were transferred from the exponentially growing pre-cultures (OD_600_ of ∼0.3) to 1 L of fresh ASW medium and cultivated at 20°C in the dark on a shaker (100 rpm). After reaching mid-exponential phase (OD_600_ of ∼0.5), sub-sampling of the initial experimental conditions was done as described below (‘T0’) and each of the cultures was split in half. While the one half was treated with the antibiotic mitomycin C (0.5 μg mL^–1^ final concentration, Carl Roth) to induce the prophages (‘treatment’), the other half served as untreated control (‘untreated control’) (Supplementary Figure 1). After 30 min of exposure to mitomycin C, both, the treatments, and controls, were centrifuged at 3,500 × *g* for 20 min at 20°C. The cell pellets were washed three times with ASW to remove the antibiotic and medium carryover before resuspension in fresh ASW. Serial filtration and sub-sampling were initiated when the surviving cells within the treatments started growing again. Additionally, the following blanks were prepared for the DOM analysis: An ultrapure water blank served as procedural blank, sterile ASW served as medium blank and sterile ASW amended with mitomycin C (0.5 μg mL^–1^ final concentration) served as mitomycin C blank. Furthermore, a physically disrupted control was prepared by growing a culture of *R. sulfidophilum* analogous to the other cultures and then succumbed to three cycles of freezing at −20°C and thawing at room temperature to completely lyse the cells (‘disruption control’). The intention was to discriminate between molecular formulae originating from the cell lysate and from the virus particles, assuming that virus infection does not change the molecular composition of bacterial cells. All blanks were incubated and treated as the culture setup.

### Sampling and Set Up of the North Sea Water Experiment

Surface North Sea water (NSW) was collected at the seaward side of Spiekeroog Island in the German North Sea in September 2020 during high tide (53°46′50.3′′ N, 7°41′34.7′′ E). The water was collected in acid-washed carboys (0.1 M HCl for 48 h). Back in the laboratory, the seawater was pre-filtered through pre-combusted 8 μm glass fiber filters (Whatman, Maidstone, United Kingdom) within 3 h after sampling to remove grazers and larger sediment particles and stored for 2 days at 4°C. Two different setups of NSW were prepared in triplicates in 1 L combusted Schott bottles (Supplementary Figure 2). The first setup consisted of natural NSW (‘untreated NSW’ setup), while the other setup of NSW was spiked with 0.22 μm filtered (Nuclepore Track-Etch Membrane, Whatman) virus-induced lysate (‘spiked’ setup) to increase a potential virus signal in the incubations. The lysate was produced by prophage induction of *R. sulfidophilum* that was analogously grown to the culture experiment and accounted for 10% of the total sample volume. Furthermore, the following blanks were prepared: an ultrapure water procedural blank, and a culture medium blank consisting of mitomycin C treated ASW. As in the culture setup, a disruption control was prepared by subjecting NSW to three freeze and thaw cycles. The untreated NSW and blanks were incubated for 2 days at 15°C at a day and night cycle of 12:12 h and light intensity of 15 μmol photons m^–2^ sec^–1^ until filtration. Within 24 h after addition of the lysate to the spiked setups, all samples were subjected to sequential filtration.

### Sequential Filtration and Subsampling Procedure

**FIGURE 1:**
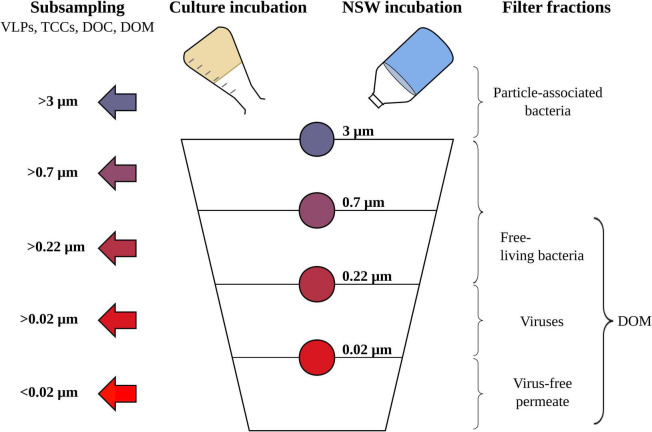
Schematic overview of the sequential filtration steps for the *Rhodovulum sulfidophilum* cultures and North Sea water (NSW) setups, including all size filter fractions (indicated by the color code) and subsampling steps (indicated by the arrows). Subsampling was done for counting of virus-like particles (VLPs) and bacterial cells (TCCs), dissolved organic carbon (DOC) quantification and the molecular characterization of dissolved organic matter (DOM).

All culture and NSW setups were sequentially filtered using acid-washed filtration towers (47 mm filter diameter, Nalgene). The following filter types (Whatman) were used: polycarbonate (Nucleopore, 3 and 0.22 μm), glass fiber filters (GF/F, 0.7 μm), and aluminum oxide (Anodisc, 0.02 μm). Filter fractions >3 μm account for a particle-associated bacterial fraction, the 0.7 μm filtrate represents the DOM fraction and the 0.22 μm filtrate accounts for the DOM and free-living bacteria fraction. The 0.22 μm filtrate encompasses the virus fraction and DOM and the 0.02 μm filtrate is the virus-free DOM (Figure 1). During filtration, a vacuum of max-200 mbar was applied to avoid breaking the cells. Pre-combusted 2.7 μm GF/D glass fiber filters (Whatman) were used as support filters. Due to supply difficulties of 3 μm glass fiber filters associated with the global coronavirus pandemic, we needed to fall back on the 2.7 μm GF/D filters as support filters that we had in stock in our lab facilities. Thus, the >3 μm particle-associated bacteria fraction in reality represents a >2.7 μm filter fraction. The filtrate of each filter fraction was collected and sub-sampled for the quantification of virus and cell numbers as well as the quantification and molecular characterization of DOM before the next filtration step. Samples for the enumeration of bacterial cell numbers and virus-like particles were fixed in 2 and 0.5% glutaraldehyde (final concentration, epifluorescence-microscopy grade, and Carl Roth), incubated in the dark for 30 min at 4°C and stored at −20 and −80°C, respectively. Aliquots for DOC quantification and DOM analysis were combined and stored in acid-rinsed PTFE bottles at −20°C until analysis.

### Quantification of Virus-Like Particles and Bacterial Cell Numbers via Flow Cytometry

Virus-like particles (VLPs) for all DOM size fractions were quantified by flow cytometry according to Brussaard et al. (2010). Viral particles were detected as SYBR Green I-stainable particles of a certain size in the >0.02 μm filter fraction. The same criterion of size was used for counting viruses in samples of the other filter fractions. However, while this definition misses a certain fraction of viruses, for example large viruses that are retained on the filter, prophages or viruses that are not efficiently stained by SYBR Green I (e.g., RNA and single-stranded DNA viruses), non-viral particles such as gene transfer agents, membrane vesicles or small cells may also be erroneously counted as viral particles (Soler et al., 2008). To account for this degree of uncertainty, we use the term VLPs when referring to virus abundances. In brief, samples were diluted with 0.02 μm-filtered TE buffer (pH 8.0, 10 mM Tris, 1 mM EDTA, Sigma-Aldrich) and stained with 0.5% SYBR Green I (final concentration, Invitrogen) for 10 min at 80°C, followed by a 5 min cooling period. Bacterial cell numbers were determined after dilution with 0.22 μm filtered PBS buffer and staining with 5% SYBR Green I (final concentration) for 30 min in the dark (Giebel et al., 2019). Multifluorescent latex beads (1 μm diameter) were added to the samples as internal standards (Polysciences Europe). Both, virus and bacterial cell samples were analyzed by an Accuri C6 flow cytometer (Becton Dickinson). For virus counts, the fluorescence threshold was set on FL1-H 450 and samples were analyzed maintaining an event rate below 1,000 events s^–1^. Final viral abundances were calculated after background correction using TE blanks (i.e., without biological sample) that were prepared and analyzed as the samples. Bacterial samples were run for 2 min at a flow rate of 14 μL min^–1^ with an event rate below 1,200 and a threshold channel set on 900. Data analysis was performed by the BD Accuri C6 software (version 1.0.264.21).

Following flow cytometric quantification, the average input of C, N, and P of VLPs and cells to the incubations was calculated. Basis for the calculations were the VLP abundances after cell lysis (>3 μm filter fraction) and literature values for the average amount of these elements per cell and virus (see Table 2). To calculate the input of the lysed cells, average values of cell numbers after virus-mediated cell lysis (>3 μm fraction) were subtracted from the averaged initial cell numbers. 50 fg of C per cell were used, following the recommendation of another study on fast-growing bacterial cultures (Wienhausen et al., 2017).

**TABLE 1:** Mass to charge ratio (*m/z*) and molecular formulae from all filter fractions that significantly (*p* < 0.05) correlated with virus abundances, detected in the *Rhodovulum sulfidophilum* culture (Culture), North Sea water spiked with *R. sulfidophilum* (NSW), prophage-induction of deep-sea sediment slurries (Bering Sea) (Heinrichs et al., 2020) and a mesocosm with a North Sea phytoplankton bloom (Mesocosm) (unpublished).

*m/z*	Molecular formula	Culture	NSW	Bering Sea	Meso-cosm	Alternative formula	PubChem match
116.0717	C5H11NO2		x	x			Valine
145.061858	C5H10N2O3	x					Glutamine
146.045878	C5H9NO4	x			x		Glutamic acid
232.11904	C10H19NO5		x	x			Homoserine
255.088748	C13H12N4O2	x			x		
271.093546	C11H16N2O6	x			x		
289.104129	C11H18N2O7		x	x	x		
315.119763	C13H20N2O7		x	x			Uridine/deoxythymidine
316.151401	C13H23N3O6	x			x		Amino acid chain (e.g., Val-Ala-Glu; Ile-Glu-Gly)
345.130351	C14H22N2O8	x			x		
386.120465	C15H21N3O9		x				
393.049687	C14H18O11S		x	x			
428.052351	C22H11N3O7		x	x			
434.131733	C28H22NO2P	x				C18H21N5O8	
447.123805	C29H20O5		x				
447.125642	C17H24N2O12		x				
449.129968	C18H26O13		x	x			Pyranoside
450.262612	C21H42NO7P	x	x	x	x		Phosphoethanolamine
455.111424	C20H25O10P		x	x			
456.047297	C23H11N3O8		x				
474.172951	C19H29N3O11		x	x	x		
482.260545	C21H41NO11	x			x		
492.183481	C19H31N3O12		x	x	x		*N*-acetyl-muramyl-L-alanyl-D-dlutamic acid
493.186787	C28H30O8		x	x			
509.286046	C28H47O4PS	x					
511.295204	C22H45NO11	x				C24H47O9P	Glycerol-3-phosphate
531.012727	C16H12N4O17		x	x			
535.032562	C17H16N2O18		x	x	x		
539.005641	C21H17O13PS		x	x			
548.209703	C22H35N3O13		x				Cell surface sugar (e.g., alpha-D-mannopyranose)
592.991116	C20H18O17S2		x				
594.988178	C19H16O20S		x				
604.26648	C33H39N3O8	x					
626.095333	C43H18NO3P		x	x		C33H17N5O9	
640.110933	C44H20NO3P		x	x		C34H19N5O9	
641.114317	C43H18N2O5		x	x			
660.605087	C41H79N3O3	x					
666.226374	C38H38NO8P	x				C28H37N5O14	
677.252374	C37H43O10P		x	x			
716.37095	C31H59NO17	x				C32H61O15P	
938.384621	C45H65NO18S	x				C37H61N7O21	*N*-acetyl-beta-D-glucosaminyl-glycopeptide

*Alternative molecular formulae are given by increasing the allowance of N and P during molecular formulae assignment compared to default settings as well as potential matches of the molecular formulae with compounds from the PubChem database.*

**TABLE 2:** Average inputs of C, P, and N in the treatments and controls of the culture experiment by the virus particles, calculated with published estimates for the element ratios (N given for viruses with an average capsid size of 50–70 nm, which is comparable to the capsid size of the *R. sulfidophilum* phage).

Element per virus particle	Average viral input in treatments (mL^–1^)	Average viral input in controls (mL^–1^)	Average input due to cell lysis (mL^–1^)
0.055–0.2 fg C^†^	2.23 × 10^6^ fg C	5.36 × 10^5^ fg C	5.83 × 10^9^ fg C
0.0078–0.02 fg N^††^	1.83 × 10^–26^ fg N	4.39 × 10^–27^ fg N	1.24 × 10^9^ fg N^‡^
0.0025–0.0074 fg P^††^	1.42 × 10^–10^ fg P	3.40 × 10^–11^ fg P	1.07 × 10^8^ fg P^‡‡^

*^†^Wilhelm and Suttle (1999), Steward et al. (2007), ^††^Jover et al. (2014); ^‡^average taken of stoichiometric ratios from Zweifel et al. (1993), Fukuda et al. (1998), and Suttle (2007); ^‡‡^average taken of stoichiometric ratios from Zweifel et al. (1993) and Suttle (2007).*

### Epifluorescence Microscopy

Representative samples for bacteria and virus counting (covering all sample types and the abundance range of the whole sample set) were additionally enumerated using epifluorescence microscopy. Samples from filter fractions >0.22 μm were centrifuged at 2,000 × *g* for 5 min to reduce the background fluorescence of cell debris. Virus filters were prepared after Suttle and Fuhrman (2010). In brief, samples were diluted using 0.02 μm-filtered PBS buffer, vacuum-filtered onto 0.02 μm Anodisc filters (Whatman), stained with SYBR Green I for 15 min in the dark and mounted onto microscopic slides with 0.1% *p*-phenylenediamine as antifade solution. Bacterial cells were filtered onto 0.22 μm polycarbonate filters (Nucleopore, Track-Etch) as described by Lunau et al. (2005) and stained with a SYBR Green I staining solution for 30 min as described by Pohlner et al. (2017). A minimum of 300 viruses and 100 bacterial cells, respectively, were counted per filter in at least 15 randomly chosen counting grids at a 1,000× magnification (Leica DMRBE Trinocular, Leica Microsystems).

### Quantification and Molecular Characterization of Dissolved Organic Matter via Fourier-Transform Ion Cyclotron Resonance Mass Spectrometry

Dissolved organic matter was concentrated and desalted via SPE using 100 mg Varian Bond Elute PPL cartridges (Agilent Technologies) as described in Dittmar et al. (2008). After gently thawing the water samples at 4°C overnight, pH of the combined DOC-DOM aliquots was adjusted to 2 using HCl (25%, p.a). Samples for DOC quantification were taken from the aliquots in duplicates and stored in pre-combusted glass vials at 4°C until analysis. Then, 50 mL of each sample were extracted with pre-cleaned PPL cartridges via gravity. The cartridges were rinsed with acidified ultrapure water (pH 2) to remove the remaining salt. Subsequently, resins were completely dried with argon gas, before the SPE-DOM was eluted with 1 mL of methanol (HPLC grade). All extracts were stored at −18°C until mass spectrometric analysis.

For FT-ICR-MS analysis, concentrations of all DOM extracts were adjusted to a DOC concentration of 2.5 ppm with a 1:1 mixture of methanol (MS grade) and ultrapure water. Carbon concentrations of the SPE-DOM extracts were determined after drying the methanol from aliquots of the SPE-DOM extracts at 50°C overnight and re-dissolution in ultrapure water of pH 2. DOC concentrations of samples and extracts were quantified using high temperature catalytic oxidation on a Shimadzu TOC-V_*CPH*_ Total Organic Carbon Analyzer equipped with an ASI-V autosampler and a TNM-1 module (Shimadzu). Analytical trueness and precision were determined using reference deep-sea water (provided by the Hansell Organic Biogeochemistry Laboratory, University of Miami) and were better than 6%. For molecular analysis, all SPE-DOM extracts were analyzed in random order on a solariX FT-ICR-MS with a 15 Tesla magnet (Bruker Daltonics) and an electrospray ionization source (ESI, Bruker Apollo II). Extracts from the culture setup were measured twice, while no technical replicates from the NSW extracts were analyzed due to time constraints. The samples were injected by an autosampler (PAL RSI CTC Analytics) into the electrospray source at a flow rate of 0.03 μL s^–1^ using ESI in negative mode. After accumulation in the hexapole ion trap for 0.1 s, negatively charged ions were transferred into the ICR cell (Infinity cell, Bruker Daltonics). For each mass spectrum, 200 scans were recorded in broadband mode using 8 mega word datasets in a mass window of 91 to 2,000 Da. To monitor instrument variability, an in-house reference DOM sample ran in between the samples. The material was collected from North Equatorial Pacific Intermediate Water from a depth of 670 m near Hawaii using the same extraction method as for the samples (Green et al., 2014). Each mass spectrum was internally calibrated with a reference mass list of known compounds that covered the targeted mass range, reducing the mass error to <0.1 ppm. Data were further processed using the software ICBM Ocean (version 1.1) (Merder et al., 2020) to remove noise, align samples along matching masses and assign molecular formulae to each detected mass. A method detection limit of 3.5 was set to separate analyte peaks from instrumental noise (Riedel and Dittmar, 2014). Sample junction was applied in fast join mode (0.5 ppm sample tolerance) and a recalibration tolerance of 0.5 ppm. Minimum signal to MDL ratio as backbone for recalibration was 1 using mean recalibration mode. Masses were aligned among samples with a 0.5 ppm tolerance to reduce random mass error. Molecular formulae were assigned according to the default settings, allowing for C_1–100_H_2–200_O_0–70_N_0–4_S_0–2_P_0–1_. These settings are adapted for natural marine and terrestrial DOM mixtures. To better reflect the vDOM and more specifically the virus-specific DOM composition in our dataset, the range of allowed P and N heteroatoms during formulae assignment was gradually increased from 1 to 3 for P and from 4 to 7 for N and compared to the dataset generated with the standard settings. To exclude unlikely formulae and double assignments, the N,S,P rule, isotope verification (1,000‰) and the homologous series network for CH_2_, CO_2_, H_2_, H_2_O, and O was applied as well as all singlet molecular formulae were removed. Molecular formulae without any oxygen atom and remaining double assignments were removed from the dataset. Based on their molar H/C and O/C ratios, their heteroatom contents, and the modified aromaticity index (AI_*mod*_) and double-bond equivalent (DBE) (Koch and Dittmar, 2006, 2016), the identified molecular formulae were categorized in operationally defined compound groups. Even though the assignment to specific molecular groups is ambiguous since behind each given molecular formula can be an unknown number of structural isomers, this categorization indicates potential structures and thus helps to identify patterns within the DOM molecular composition. Defined groups were aromatics (AI_*mod*_ > 0.5), highly unsaturated compounds (AI_*mod*_ < 0.5, H/C ≤ 1.5), unsaturated compounds (1.5 ≤ H/C ≤ 2, O/C < 0.9), unsaturated compounds with N (1.5 ≤ H/C ≤ 2, O/C < 0.9, N > 0) and saturated compounds (DBE = 0). In the culture setup, only molecular formulae detected in both analytical replicates were considered for further data analysis, while only molecular formulae which were present at least three times in the whole dataset were included in the NSW dataset. Molecular formulae in the samples were normalized to the sum of the total FT-ICR-MS signal intensities. Prior to multivariate statistical analysis, all molecular formulae that were also present in the sterile blanks were removed by subtracting their normalized relative peak intensities from those of the samples. The rather strict removal of contaminations was done to ensure that molecular formulae assigned to viruses were no contaminations.

### Statistical Analysis

All statistical analyses were performed on normalized DOM data in R (version 3.6.2, RCore Team, 2019) using the RStudio interface (version 1.4.1717, RStudio Team, 2021). First, the dataset was Hellinger-transformed to give less weight to rare formulae (Ramette, 2007). The molecular dissimilarity between samples was calculated using Bray–Curtis dissimilarity analysis (Bray and Curtis, 1957) and visualized using the package ‘*pheatmap*’ (Kolde, 2019). To further reveal patterns in the DOM composition, principal coordinate analysis (PCoA) was performed on Bray–Curtis dissimilarity matrices using the package ‘*stats.*’ Weighted averages for the relative abundance of the molecular classes and meta-data collected during the experiment were calculated considering the peak intensity of each molecular formula. Only selected parameter that significantly contributed to the variability among the DOM samples (permutational multivariate analysis of variance with 9,999 permutations, *p* < 0.05) were fitted to the PCoA scores using the ‘*envfit*’ function of the *vegan* package (version 2.5-6, Oksanen et al., 2019). To identify molecular formulae that could be related to viruses, the relative peak intensities of the DOM dataset were correlated with the corresponding VLP abundances by Spearman rank-order correlation using the package ‘*tidyverse*’ (Wickham et al., 2019). Only molecular formulae with an adjusted *p*-value < 0.05 and a Spearman’s ρ of >0.5 were considered in the analysis. The robustness of the correlation analysis was tested by running the correlations with a randomized DOM data set using 100 permutations without replacement.

## Results

### Growth and Substrate Utilization in the Culture Set-Up

**FIGURE 2:**
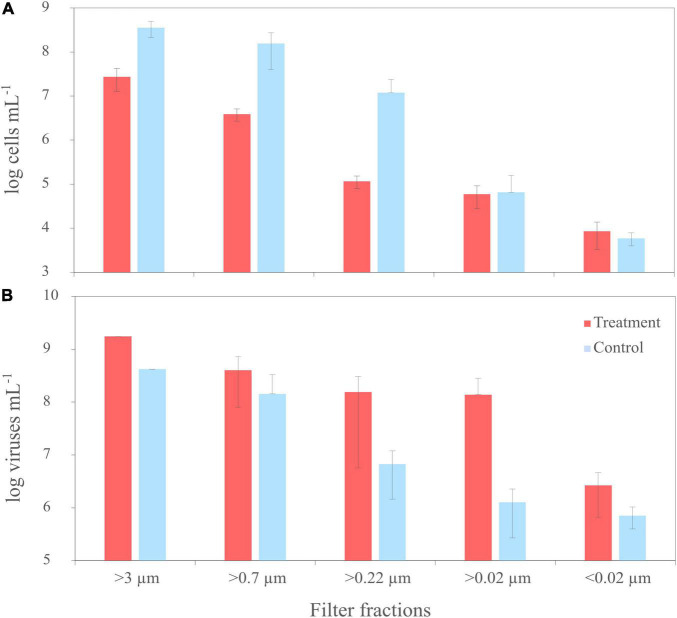
Trends of cell numbers **(A)** and virus abundances **(B)** of the mitomycin C treated (‘treatment’) and untreated cultures (‘untreated’) of *R. sulfidophilum* during sequential filtration through filters with various pore sizes. Biological replicates are averaged (*n* = 3) and standard deviations are indicated by the error bars. Virus counts from two treatments and untreated controls of the filter fraction >3 μm were not measured due to loss of samples during preparation.

Pure cultures of *R. sulfidophilum* were sampled for virus and bacterial counts at the start of the experiment (T0) and after each filtration step. Before filtration (T0), average cell numbers of the treatments, untreated controls and the disruption control ranged around 1 × 10^8^ cells mL^–1^ ± 3 × 10^7^ cells mL^–1^ (*n* = 3 ± standard deviation). While the cells in the controls continued growing by a factor of 2.5 until the start of the filtration, cell numbers in the mitomycin C treatments decreased by one order of magnitude as result of the virus-mediated cell lysis. This was also visible in an increased scattering of the fluorescence signal and a shift of the culture signal from the high nucleic acid toward low nucleic acid region in the cytograms, indicating the disintegration of the cells (Supplementary Figure 3). Accordingly, cell numbers of the treatments were lower than those of the corresponding controls in all filter fractions (Figure 2A).

At the start of the experiment (T0), numbers of virus-like particles (VLPs) exceeded bacterial cell counts about one order of magnitude. Until filtration, VLPs in the treated samples increased by a factor of 1.6 to 2 × 10^9^ VLPs mL^–1^ (*n* = 1) due to the prophage induction. Accordingly, virus abundances were higher in all samples of the treatments than in the controls, in the smaller filter fractions up to two orders of magnitude (Figure 2B). The induction worked for all three treatments, even though the degree of induction differed between the treatments. In one of the replicates, cell numbers and virus abundances were one order of magnitude lower compared to the other replicates. Due to the high variability between the replicates and the missing samples of the >3 μm filter fraction, cell and virus abundances were not significantly different (Welch Two Sample *t*-test).

The formation of cell aggregates and autofluorescence properties of undefined components of mitomycin C in the virus-induced incubation setups caused high background fluorescence. This can potentially lead to overshadowing of cells and virus-particles or elsewhere to counting of false positives, both resulting in erroneous virus or cell numbers. Thus, samples representative for the whole dataset were additionally counted using epifluorescence microscopy to validate the cell and virus abundances analyzed by flow cytometry. In linear regression analysis, the numbers for virus (*n* = 10, *p* < 0.001, *R*^2^ = 0.91) and cell abundances (*n* = 11, *p* < 0.001, *R*^2^ = 0.9136) determined by both methods were highly correlated (Supplementary Figure 4).

As most of the DOC in the samples accounted for the culture amendments, DOC concentrations of the culture setup were excluded from the analysis. Instead, concentrations of the solid-phase extractable DOC (SPE-DOC) of a representative range of samples were quantified, since the substrate was partially removed by washing and is generally not well extracted by SPE. Average SPE-DOC concentrations of the cultures were with 25.0 μmol L^–1^ ± 11.9 μmol L^–1^ (*n* = 4, ± standard deviation) marginally higher in the treatments than in the controls 22.9 μmol L^–1^ ± 12.0 μmol L^–1^ (*n* = 7) and decreased slightly in smaller size fractions.

### The Molecular Dissolved Organic Matter Composition of the Culture Setup

**FIGURE 3:**
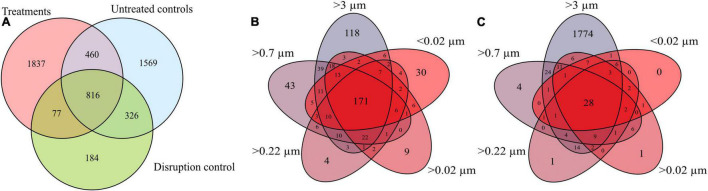
Venn diagram comparing all molecular formulae detected in all replicates and filter fractions of the treatments, controls and the disruption control **(A)**. Comparison of the average molecular formulae of each filter fraction of the treatments **(B)** and untreated controls of the *R. sulfidophilum* culture **(C)**.

**FIGURE 4:**
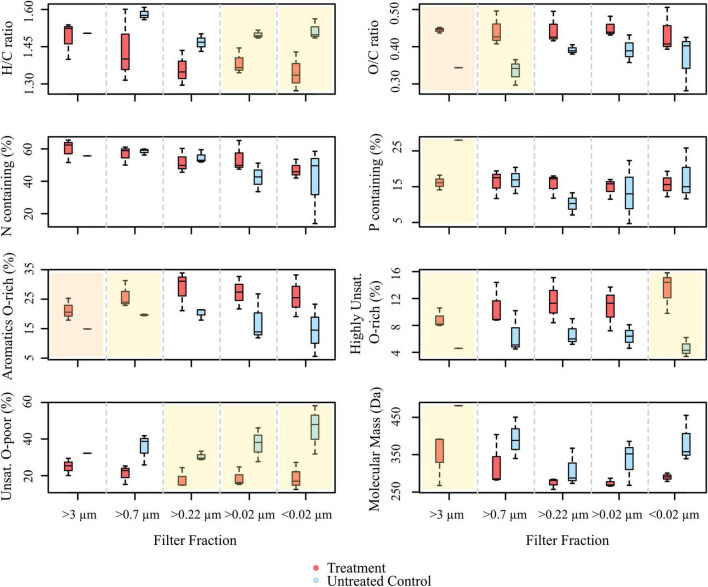
Boxplots showing the molecular compositions of signal intensity-weighted averages of various compound groups, comprised from all masses with assigned molecular formulae detected in the treatments and controls of the culture experiment (ANOVA with Tukey HSD test, *n* = 1 for >3 μm fraction of the control, *n* = 3 for the rest. The overlay in yellow indicates significant differences between treatment and control (*p* < 0.05), panels overlain in orange represent highly significant differences (*p* < 0.005).

In total, 5,269 DOM molecular formulae were assigned in the culture experiments. Overall, we detected 3,190 individual masses with assigned molecular formulae in the treatments and 3,171 in the controls of the culture incubations, including all filter fractions and replicates (Figure 3A). Of these identified formulae, 40% (i.e., 1,276 molecular formulae) were shared between treatments and controls, most of which were saturated and unsaturated compounds with high *H*/*C* ratios (Figure 4). The disruption control shared more formulae with the untreated controls than with the vDOM of the treatments. Comparing the bulk molecular characteristics of all filter fractions, the disruption control showed high *H*/*C* ratios and high percentages of unsaturated compounds, while the virus-induced treatments were more composed of molecular formulae with higher *O*/*C* ratios (>0.40) and percentages of aromatic and highly unsaturated compounds (Figure 4). Interestingly, the average amount of formulae containing P- and N-heteroatoms was similar in the treatments and controls.

The different size fractions of one replicate partially differed in their composition, a trend which was more pronounced in the treatments. Except for the >3 μm filter fraction, the size fractions of the untreated culture shared most of the detected molecular formulae (Figure 3C). The treatments on the other hand shared on average 42% (i.e., 171 molecular formulae) of all detected molecular formulae (Figure 3B). In the different DOM size fractions, the intensity-weighted average molecular masses marginally decreased in the smaller size fractions of the treatments, while there was a distinct shift toward lower masses in the controls (Figure 4).

**FIGURE 5:**
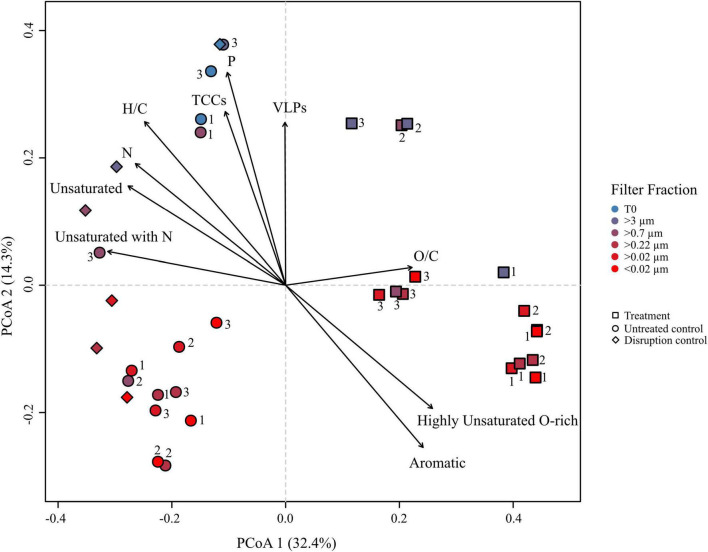
Principal coordinate analysis (PCoA) based on Bray–Curtis dissimilarities of the relative abundances of molecular formulae from the *R. sulfidophilum* culture incubations. Squares indicate mitomycin C treated cultures (‘treatments’), circles the untreated controls and diamond shapes the disruption controls. Each sample was filtered over several filters as indicated by the color code. Biological replicates are indicated by the numeration 1–3. Significant experimental meta-data and molecular parameters (*p* < 0.05) were fitted to the ordination (black arrows).

Principal coordinate analysis (PCoA) was performed on the DOM dataset of the culture to visualize dominant patterns and trends. The PCoA graphically describes the relationship between different samples based on the two major axes of variation. Samples plotting closer together in the PCoA have a more similar molecular DOM composition than samples stronger separating from each other. The difference in the molecular composition between controls and treatments explained the largest variability in the dataset (PCoA 1 = 32.4%) (Figure 5), while the second axis largely represents a shift in the molecular composition following filtration (PCoA 2 = 14.3%). The larger filter fractions (T0, >3 and >0.7 μm) of both, controls and treatments distinctly separated from the smaller filter fractions. Even though the smaller filter fractions clustered closer together, the molecular composition between the fractions of the same replicate still differed from each other, exceeding the analytical variance in most cases. The treatment with the lower cell and virus abundances clustered slightly apart from the other treatments, indicating a slightly different composition. However, the overall molecular composition of the different filter fractions of the treatments was more similar to each other, while the controls separated more pronounced according to the pore size of the filters, in accordance with Figures 3B,C. Interestingly, the disruption controls grouped with the untreated controls and not with the treatments. The treatments correlated with the relative abundance of aromatic and highly unsaturated, oxygen-rich compounds. The bulk molecular composition of the untreated controls was related to the relative abundance of unsaturated and N-containing compounds. Consistently with the cell and virus counts, the larger fractions were more influenced by VLP and cell abundances and interestingly also P-bearing compounds. A Bray–Curtis dissimilarity analysis confirmed the PCoA results (Supplementary Figure 5).

### Growth in the North Sea Water Setup

**FIGURE 6:**
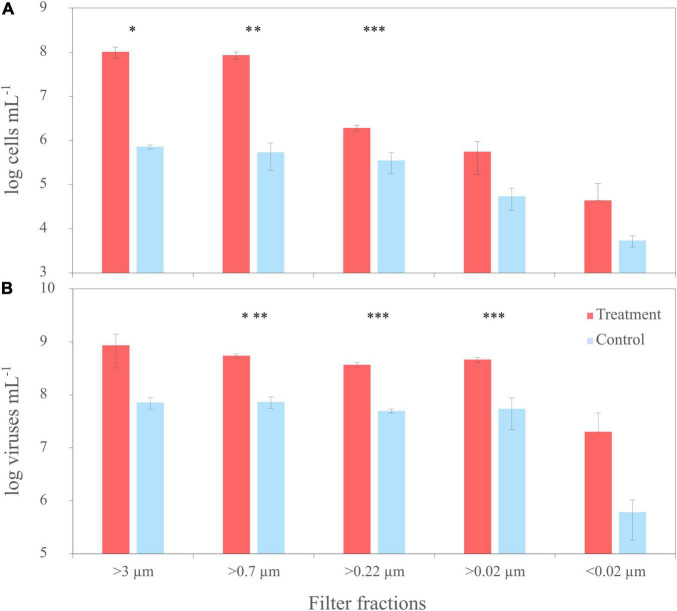
Trends of cell numbers **(A)** and virus abundances **(B)** of the sequential filtration of untreated North Sea water (NSW) through filters with various pore sizes (‘untreated’) and NSW spiked with a cell lysate of *R. sulfidophilum* (‘spiked’). Biological replicates are averaged (*n* = 3) and standard deviations indicated by the error bars. Asterisks indicate significant differences between treatments and controls (*p < 0.05, **p < 0.01, ***p < 0.005).

To compare the virus DOM signature that derived from a single bacterial strain with a potential virus signature in a complex natural microbial community, the same filtration experiment as performed with the culture was repeated using untreated NSW. NSW was furthermore spiked with the lysate from *R. sulfidophilum* to increase the virus signal. At the start of the experiment (T0), average cell abundances in the spiked and untreated NSW setup ranged between 2 and 3 × 10^6^ cells mL^–1^ ± 2 and 7 × 10^5^ cells mL^–1^ (*n* = 3 ± sd.), respectively (Figure 6A). Bacterial cell abundances of the untreated NSW gradually decreased in the smaller size fractions. Until the start of filtration, cell numbers in the spiked NSW setups increased to roughly 1 × 10^8^ cells mL^–1^ ± 3 × 10^7^ cells mL^–1^, indicating that the NSW microbial community may have grown on the culture lysate. The differences between controls and treatments were statistically significant. With an average of 8 × 10^7^ VLPs mL^–1^ ± 2 × 10^7^ VLPs mL^–1^, initial virus abundances were on average 2.9 times higher than cell abundances in the untreated and spiked NSW (Figure 6B). While virus abundances marginally decreased in smaller filter fractions of the untreated controls, VLPs of the spiked samples increased to 9 × 10^8^ VLPs mL^–1^ ± 5 × 10^8^ VLPs mL^–1^ and highly significantly exceeded those of the untreated NSW about one order of magnitude in all filtration steps (*p* < 0.005).

### Molecular Dissolved Organic Matter Characterization in the North Sea Water Setup

The average DOC concentration of the untreated NSW of 95.5 μmol L^–1^ ± 9.6 μmol L^–1^ (*n* = 11, ± sd.) was slightly higher for the spiked samples with 108.5 μmol L^–1^ ± 12.6 μmol L^–1^ (*n* = 9), taking into account all replicates and filter fractions. The average extraction efficiency of the untreated NSW samples was 40.7% ± 3.6% (*n* = 9), while the extraction efficiency in the spiked samples was 14.1% ± 10.5% (*n* = 8). Overall, the processing of the entire NSW DOM dataset resulted in 4,826 different molecular formulae. In total, 3,076, 4,656, and 4,031 individual molecular formulae were detected in the entire subset of spiked, untreated NSW and the disruption control, respectively. All filter fractions of the untreated NSW and disruption control shared 94.5 and 88% of formulae with the spiked samples. The molecular composition of the disruption control was almost identical with the untreated NSW.

Principal coordinate analysis and Bray–Curtis dissimilarity analyses revealed similar trends in the DOM composition of all samples with those of the culture setup (Supplementary Figures 6, 7). In the PCoA ordination plot, all spiked samples separated from the untreated NSW samples and disruption control on the first major axis of variation (PCoA 1 = 48.2%). On the second axis, the samples separated according to the pore size of the filters (PCoA 2 = 13.8%). However, the samples clustered independently of replicates and filter fractions, indicating that the molecular DOM composition of the individual filter fractions was more similar compared to the culture setup. This is supported by the higher overlap of shared formulae between corresponding filter fractions of replicates and by a lower Bray-Curtis dissimilarity range compared to that of the culture experiment (Supplementary Figures 5, 7). When comparing the intensity weighted averages of all samples, the bulk molecular compositions of the replicate filter fractions were quite similar. The samples spiked with culture lysate were significantly correlated to the relative abundance of DOM molecular formulae categorized as unsaturated and oxygen-rich compound groups. Furthermore, they were correlated to the relative abundance of molecular formulae containing high amounts of N- and P- heteroatoms as well as to high VLP and cell abundances (*n* = 39, *p* < 0.0001). In contrast, the untreated NSW correlated with saturated, oxygen-poor highly unsaturated and aromatic compounds (*n* = 39, *p* < 0.0001). Other pronounced differences between spiked and untreated samples were the higher intensity-weighted contents of P- and N-bearing molecules and higher occurrences of unsaturated compounds with and without N. In general, the abundance and intensity of molecular formulae deriving from the lysate was approximately 30% less in the spiked samples compared to the culture samples.

### Deciphering the Virus Signal Within the Culture and the North Sea Water

To extract molecular formulae within the culture DOM pool that derived from viruses, we correlated all molecular formulae of the dataset with the corresponding virus abundances using Spearman rank order correlation. Out of the 5,269 formulae of the culture setup 16 were significantly correlated with virus abundances (*p* < 0.01) and were especially detected in the treatments and larger filter fractions (Table 1). Roughly two thirds of these molecular formulae were categorized as unsaturated compounds with and without N, and 94 and 25% contained N and P-heteroatoms, respectively. Overall, they were recovered throughout the whole DOM dataset of the culture experiment, accounting for an average of 1.4% of molecular formulae and 1.2% of the total intensities of the samples. A linear regression analysis comparing the sum of intensities of the correlated molecular formulae with the virus numbers showed a general increase of signal intensities with increasing virus abundances (*R*^2^ = 0.47, *p* < 0.001). The increasing allowance for P and N heteroatoms during molecular formulae assignment in the DOM processing tool ICBM Ocean resulted in an increase of the average amount of N from 1.69 to 2.44 without changing the percentage of the overall molecular formulae with N-heteroatoms. Formulae containing P increased up to 14% by this data processing procedure. Some of the correlated molecular formulae matched with those of typical bio-molecules such as amino acids, peptides or saccharides in the PubChem database. Moreover, other formulae also matched with structural components of the bacterial peptidoglycan cell wall and cell membrane (e.g., phospholipids, N-muramic acid compounds, and derivatives) (Table 1).

In the NSW dataset, the Spearman rank correlation identified 26 molecular formulae that significantly correlated with virus abundances (*p* < 0.05) (Table 1). Most of them (81%) derived from spiking. Accordingly, intensities and abundances of the correlated molecular formulae were much higher in the spiked samples, especially in the larger filter fractions. On average, the correlated formulae accounted for 4.6% of the total intensity, 1.8% of all formulae of the spiked samples, and <0.01% in the untreated NSW. The average molecular composition of the correlated molecular formulae was defined by a high *O*/*C* ratio (0.54), high percentages of N- and P-heteroatoms (65 and 23%, respectively) and by highly unsaturated (46%) and unsaturated compounds (27%). Again, by adjusting the default settings for the formulae assignment in ICBM Ocean, the number of molecular formulae containing P and N increased by <10%. Some correlated molecular formulae in the PubChem database matched amino acids, components of the DNA double helix (e.g., intermediate pyrimidine) or sugars associated to bacterial cell walls and cell surfaces (Table 1).

### Recovery of Virus Signals Within Other Datasets

We tested if the correlated molecular formulae of the culture and NSW setups could be also detected in other virus containing DOM datasets from different environmental backgrounds. The first dataset originated from incubations of deep-sea sediments retrieved from the Bering Sea (Heinrichs et al., 2020). In these sediments, prophages of the benthic microbial communities were induced using mitomycin C (Paul, 2008). One third of the correlated molecular formulae from the culture setup and half of the molecular formulae from the NSW setup were also found in the DOM dataset from the Bering Sea experiment (Table 1). Overall, the correlated molecular formulae of the culture setup and the NSW setup accounted for <0.1% of all molecular formulae and total intensities, respectively. The highest recovery of formulae in terms of abundance and intensities were found in the incubation with the highest virus numbers due to prophage induction.

The second DOM dataset was derived from an indoor mesocosm experiment, following the course of a diatom and subsequent *Phaeocystis* sp. blooms after inoculation with a natural microbial community from the North Sea (Mori et al., 2021). In four replicate mesocosm tanks (Gall et al., 2017), the succession of the phytoplankton blooms and following bacterio- and virioplankton blooms as well as the molecular DOM composition were monitored (unpublished). Less than 20% of the correlated molecular formulae of the NSW incubations and of the culture setup were recovered throughout the entire mesocosm DOM dataset (Table 1). While the correlated molecular formulae of the culture setup were negligible and only sporadically found in the mesocosm samples, the molecular formulae of the NSW setup accounted for 0.02–0.04% of the total intensity and the total number of detected molecular formulae in all four mesocosms.

## Discussion

So far, viruses were found in every investigated habitat of the marine environment. Due to their small size, most viruses fall under the oceanographic size class definition of DOM and are thus integral part of almost all marine DOM samples. Aim of this study was to investigate if a DOM signature derived from virus particle components can be detected within the analytical window following routine sample preparation and state-of-the-art DOM mass spectrometric analysis. For this purpose, the molecular DOM composition of a lysogenic bacterial culture and of NSW containing a natural microbial community were analyzed.

### The Virus Signal Can Hardly Be Detected in the Virus-Induced Dissolved Organic Matter Pool of the Culture

Spearman rank order correlation analyses extracted a small subset of DOM molecular formulae from the datasets that were positively correlated with virus abundances of the NSW and culture setup, respectively. A linear regression confirmed a relationship between virus abundances and intensities of the correlated molecular formulae (*R*^2^ = 0.47, *p* < 0.001). Even though this subset of molecular formulae only accounted for a minor fraction of both DOM datasets, their attribution to virus particle abundances is yet very robust due to the (highly) significant correlation (*p* < 0.05 and *p* < 0.01). All molecular formulae correlated to the virus particles were highly enriched in N and P, compared to the bulk DOM composition, consistent with the bulk composition of viruses, which are mainly made up by amino acids and peptides (∼76%), nucleotides (18% DNA) and sometimes glycoproteins (Knight, 1975). The overall molecular composition and the match of the correlated formulae with compounds such as peptide chains and DNA fragments (e.g., uridine or deoxythymidine) indeed point toward a potential viral origin. However, some of the correlated molecules might also originate from the cell lysate of *R. sulfidophilum*. The potential match of the formulae with components that are associated with bacterial cell walls (e.g., cell surface sugars of Gram-negative bacteria, glycerol ester, muramic acid complex; Table 1) implies that they could also stem from processes related to viral activity (Zheng et al., 2021), underlining the challenge to detect the building blocks of virus particles using FT-ICR-MS (Hawkes et al., 2016). However, it should be kept in mind that matching the correlated molecular formulae with compounds from the database is ambiguous: First, an enormous number of different structural isomers may exist behind each molecular formulae detected by FT-ICR-MS (Zark et al., 2017; Hawkes et al., 2018). Second, several entries can be found in the PubChem database for most sum formulae. Third, not all possible compounds are listed in the database.

A rough calculation based on literature values for C, P, and N showed that virus particles introduced one order of magnitude more C, P, and N to the incubations of the induced cultures, compared to the untreated controls (Table 2). Despite the difference in the input of virus-derived elements to the DOM pool, the amount of P and N-containing molecular formulae was not significantly higher in the treatments (Figure 4), again indicating that the influence of virus particles on the DOM pool is barely detectable when using routine settings. Even though viruses are enriched in N and P compared to the average content of a microbial cell or the canonical composition of phytoplankton cells (Suttle, 2007; Klausmeier et al., 2008; Jover et al., 2014), the elemental input by viruses vanished behind the estimated input of C, N, and P from the lysed cell in the treatments on the basis of a carbon content of 50 fg cell^–1^ for large, fast-growing bacteria (Simon and Azam, 1989) (Table 2). Thus, the observed correlation of N- and P-containing molecular formulae in samples with high virus numbers in the PCoA (Figure 5) could rather stem from the bacterial cell debris than from the virus particles themselves.

### The Detection of the Virus Signal Is Limited by the Analytical Window

The detection of virus derived components via our technique comes along with certain analytical challenges. First, no analytical technique is so far capable to detect the whole spectrum of DOM molecules due to the enormous molecular diversity of DOM (Zark et al., 2017; Hawkes et al., 2018). Second, the sample preparation and analysis both select toward a certain range of molecules (Hertkorn et al., 2013; Hawkes et al., 2016; Raeke et al., 2016). In previous studies on growth experiments with various microorganisms (Longnecker et al., 2015; Wienhausen et al., 2017; Noriega-Ortega et al., 2019), the extraction efficiencies were in the lower range of extractable DOM by the cartridge (e.g., approx. 40% at the stationary phase). This indicates that a certain fraction of freshly produced organic matter such as cellular material and individual fragments of virus particles are poorly retained by SPE. We cannot evaluate the effect of extraction efficiencies, as those were not calculated for the culture setup due to missing DOC values.

While the detection of molecules recovered by SPE by the mass spectrometer further depends on the ionization efficiency of the individual compounds, not further specified elements within the sample matrix moreover can alter the ionization efficiency of target analytes, either causing an ion suppression or enhancement (Brown and Rice, 2000; Bercovici et al., 2022). This so-called matrix effects potentially lead to an under-representation or absence of compound classes with low abundance or ionization efficiencies in the resulting mass spectra. The preferential ionization of compounds within the natural DOM background of the NSW and the high concentration of lysis products of the bacterial culture, both could have contributed to a suppression of the virus compounds (Kido Soule et al., 2015; Lucas et al., 2016). Thus, the virus signal might further be hidden behind the natural DOM background of marine samples or by lysis by-products.

### The Detection of a Virus Signal in Dissolved Organic Matter Datasets From Natural, Complex Environments

To compare the virus signal stemming from a single bacterial strain with a potentially more complex signal from a natural microbial community, the filtration experiment was repeated with NSW. Bacterial cell abundances of the NSW setup were in the typical range for coastal regions of the North Sea (Rink et al., 2011), while VLPs were similar like numbers of other highly productive sampling locations, close to the West Frisian Island chain (Winter et al., 2004; Parada et al., 2008). The molecular DOM composition correlated with aromatic, highly condensed compounds, probably due to the input of terrestrial material at the sampling site (Seidel et al., 2015), while the addition of culture lysate in the spiked NSW setup led to a detectable change of the molecular DOM composition with a higher occurrence of P- and N-rich, unsaturated components. However, the lower abundance and relative intensity of molecular formulae stemming from the lysate compared to the expected abundance indicates the growth of heterotrophic microorganisms on the lysates in accordance with the surge of bacteria cell counts (Figure 6). Moreover, components of the lysate might have been less favorable ionized in the presence of the natural DOM background of the NSW (Bercovici et al., 2022).

The DOM molecular formulae that significantly correlated with the virus abundances were exclusively derived from spiking with the bacterial culture lysate. Furthermore, no significant correlation with the virus abundances was found in a reduced NSW dataset containing only the molecular formulae from the untreated NSW setup. This was probably because the virus abundances in the range of 10^7^ mL^–1^ were too low in the untreated NSW to be detected. In contrast, VLPs were as high as 10^8^ and 10^9^ mL^–1^ in the spiked NSW and culture experiment, respectively, probably exceeding a threshold above which components derived from virus particles are detectable, which is in accordance with another study, where viruses were found to contribute to a considerable part of the dissolved organic P and N pool when they exceed certain abundances (Jover et al., 2014).

Molecular formulae correlating with virus abundances could be recovered in both previously published DOM datasets containing virus lysis events. A larger subset of these formulae was recovered in the Bering Sea dataset than in the mesocosm dataset. This could have been partially due to the generally higher VLP numbers (∼10^10^ VLPs mL^–1^) and usage of prophage induction via mitomycin C in the Bering Sea incubations (Heinrichs et al., 2020). While the prophage induction in this incubation created a mass lysis event accompanied by the release of large numbers of virus particles, the mesocosms were likely dominated by continuous lysis events during the phytoplankton blooms (Suttle, 2005; Bartlau et al., 2021), enabling the detection of components likely derived from virus particles or cell lysis. Furthermore, the degradation of organic material was most likely lower in the deep-sea sediments compared to the faster turnover rates of the newly available substrates by the microbial community during the phytoplankton bloom (Jørgensen and D’Hondt, 2006; Buchan et al., 2014; Imachi et al., 2020). The deep-sea sediment incubations contained copiotrophic bacterial groups such as *Gammaproteobacteria* and *Flavobacteriia*, likely capable of quickly degrading virus-derived components, these groups were mainly targeted by the prophage induction treatment (Pernthaler and Amann, 2005; Fuhrman et al., 2015; Heinrichs et al., 2020). In contrast, viruses in the euphotic zone have turnover times from hours to days (Matteson et al., 2012; Lara et al., 2017), and their molecular fragments that rather fall in the targeted size range of the FT-ICR-MS are assumed to be quickly taken up by heterotrophic microorganisms (Björkman et al., 2000; Jover et al., 2014; Dell’Anno et al., 2015). This could further explain the observation that mainly formulae matching with hardly degradable cell wall derivatives were recovered in the mesocosm experiment (Middelboe and Jørgensen, 2006; Hach et al., 2020). Our results indicate that molecular formulae attributed to virus abundances via correlation may be universally detected in marine DOM datasets containing an active virus community using standard analysis routines. However, results may vary depending on the overall virus abundance, overall productivity, and the respective DOM background.

### Future Perspectives for the Detection of a Virus Signal

Generally, viruses or virus-derived components can be detected by mass spectrometry. Using different ionization methods and mass spectrometers, specific proteins were targeted such as the capsid protein (Colquhoun et al., 2006; Murray et al., 2006) or glycoproteins of enveloped viruses (Kim et al., 2001), individual subtypes of respiratory viruses were differentiated by the detection of signature peptides (Nguyen and Downard, 2013) and even intact virus particles were detected (Siuzdak et al., 1996; Fuerstenau et al., 2001; Dominguez-Medina et al., 2018). While most of these studies were done in clinical microbiology for diagnostic purposes of pathogenic viruses (Calderaro et al., 2014; Ye et al., 2019), some studies were conducted on marine cyanophages (Sabehi et al., 2012; Enav et al., 2018). Soft ionization techniques such as ESI enable the detection of whole virus particles, as they leave the protein structure of the virus capsid intact during injection into the mass spectrometer (Snijder et al., 2013). However, our standard ESI settings are optimized for single charged molecules and our mass window is too low (<2,000 Da) to detect intact virus particles or proteins. Multiple charging the capsid protein would allow to detect larger fragments or even intact virus particles via ESI-FT-ICR-MS (Dominguez-Medina et al., 2018), while a pure virus extract devoid of any cultural or natural DOM background would allow to give a deeper insight into a virus signature using our standard setting. In this study, however, it was impossible to prepare a pure virus extract due to the lack of a suitable organic carbon free technique to concentrate viruses. Common isolation techniques for virus particles such as cell sorting via flow cytometry, ultrafiltration (e.g., Amicon centrifugal filters, Merck; Vivaflow cassette, Sartorius) or density gradient ultracentrifugation all involve organic carbon components (e.g., glycerin coated filter devices, precipitation via polyethylene glycol, and sucrose/iodixanol gradients), rendering it impractical for the highly sensitive analysis via FT-ICR-MS. Moreover, samples may partially still contain debris material derived from the sample matrix depending on the size cut-off. A suitable isolation technique, free of organic carbon contaminants would allow analyzing a potential virus DOM composition in the absence of a complex sample matrix and might increase insights into a typical virus fingerprint.

### Molecular Dissolved Organic Matter Signatures of Infected Cells Differ From Uninfected Cells

Initially, the disruption control of both experiments served the purpose to separate the molecular formulae stemming from the cell lysate from those of the virus particles, as we assumed that the DOM composition of physically disrupted cells was comparable to the one originating from viral lysis. However, the DOM analysis revealed that the molecular composition of the disrupted control was more similar to the untreated controls in both experiments than to that of the infected culture (Figure 5). A microbial cell undergoing viral infection changes its metabolic activity with impacts even on ecosystem level (Ankrah et al., 2014; Rosenwasser et al., 2016; Zimmerman et al., 2020). The virocell concept, coined by Forterre (2013), refers to the cellular state of an infected host cell whose functions are redirected from host’s pathways for cellular replication toward virus particle production (Rosenwasser et al., 2016). Changes encompass metabolic fluxes (e.g., increases of nutrient uptake rates), gene expression (e.g., toward nucleotide biosynthesis), and the inventory of host cell structures, all in favor of the synthesis of virus building blocks (Zeng and Chisholm, 2012; Ankrah et al., 2014; De Smet et al., 2016; Waldbauer et al., 2019). Moreover, viruses may introduce new auxiliary metabolic genes (AMGs) to expand the hosts metabolism to support virus progeny generation, for example genes involved in the pentose phosphate pathway for the biosynthesis of viral nucleic acids (Thompson et al., 2011). The physiological differences between an infected cell and their uninfected counterparts can be detected on a transcriptional level (Morimoto et al., 2018), and also affect the lysate composition. The vDOM composition across different studies and source organisms was reported to have a distinct molecular signature differing from uninfected cells (Ankrah et al., 2014; Zhao et al., 2019; Heinrichs et al., 2020), and is especially enriched in amino acid and proteinaceous material (Middelboe and Jørgensen, 2006; Ankrah et al., 2014; Xiao et al., 2021). The differences in the composition of vDOM compared to other lysis mechanisms such as sloppy feeding, DOM exudation or mechanical lysis showed that the molecular changes that infected cell undergo can be detected using liquid chromatography-tandem mass spectrometry (Ma et al., 2018). Our results suggest that the metabolic changes bacterial cells undergo during virus infection can be also detected using FT-ICR-MS.

### Integration of the Results in the Ecological Context

In the last decades, the role of viruses as major drivers for microbial ecology and biogeochemical cycles in the ocean became increasingly apparent (Bergh et al., 1989; Wilhelm and Suttle, 1999; Zimmerman et al., 2020). Every year, up to ∼10^32^ prokaryotic cells are infected by viruses in the ocean (Suttle, 2007), releasing an estimated 150 Gt of C, 27.6 Gt of N, and 4.6 Gt of P to the environment (Wilhelm and Suttle, 1999; Suttle, 2005; Lara et al., 2017). As the released material is essential for microbial growth, the composition, turnover, and fate of vDOM derived from different marine organisms was studied with growing interest (Ankrah et al., 2014; Ma et al., 2018; Zhao et al., 2019; Heinrichs et al., 2020; Kuhlisch et al., 2021; Zheng et al., 2021). Similar to the findings of these studies, the cell lysis of *R. sulfidophilum* introduced a mixture of aromatic, oxygen-rich and unsaturated compounds that were enriched in N- and P-heteroatoms to the incubations.

However, it was not addressed in the previous studies if a fraction of the vDOM could stem from the virus particles themselves, especially the P-(Heinrichs et al., 2020) or N-rich formulae (Zhao et al., 2019; Zheng et al., 2021). The total inventory of viruses on Earth is estimated to hold 2.9 Pg of C, 0.96 Pg of N, and 0.36 Pg of P (Jover et al., 2014; Cobián Güemes et al., 2016). The elements and compounds that are released from the virus biomass upon decomposition were shown to be an important substrate and quickly turned over (Bratbak et al., 1994; Dell’Anno et al., 2015), and may especially be an essential source of labile material in oligotrophic or otherwise nutrient-limiting habitats such as in the deep sea (Jiao et al., 2010; Engelhardt et al., 2014; Jover et al., 2014; Heinrichs et al., 2020). Even though viruses outnumber prokaryotes by at least one order of magnitude across diverse habitats (Wigington et al., 2016), their contribution to the global organic carbon pool is thought to be little compared to prokaryotes due to the carbon content of a single virus particle (Jover et al., 2014; Bar-On and Milo, 2019). However, due to their fast turnover and the enrichment of N and P compared to microbial cells, virus particles may play an important role for labile DOM pool and element fluxes, especially the dissolved organic phosphorus pool (Paul et al., 1991; Bratbak et al., 1994; Cobián Güemes et al., 2016). It is estimated that virus particles can comprise >5% of the total marine dissolved organic P and N pools in some locations (Jover et al., 2014). While the results of our experiment suggest that DOM released by viral lysis of bacterial cells contributes more substantially to the detected DOM signature, still a small imprint of virus particles on the vDOM composition (<1%) was traceable by untargeted ultrahigh-resolution mass spectrometry. The detection of a molecular signature of viruses is challenging as the elemental contribution of virus particles in natural samples may be too low due to their small molecular weight, their fast turnover and a high presence of a natural background. However, due to their unique elemental composition, their high numbers and activities, viruses are a natural part of DOM and undoubtedly influence the overall composition, bioavailability and cycling of DOM in the ocean, as underlined by the results of this study.

## Conclusion

Using routine sample preparation and state-of-the-art DOM analysis, a small subset of molecular formulae was significantly correlated to virus abundances derived from a culture lysate. This subset of molecules was enriched in N- and P-heteroatoms and accounted for <1% of molecular formulae and <2% of intensities of the whole dataset. However, these formulae could not be attributed unambiguously to virus particles but could also be related to byproducts of the co-occurring virus-mediated cell lysis. Despite being an integral part of basically all natural seawater samples, virus particles themselves did not significantly contribute to the measured DOM signature of natural seawater using our standard procedures. The individual components of the virus biomass may not be well recovered using our SPE technique and FT-ICR-MS, especially in the presence of a complex sample matrix (Hertkorn et al., 2013; Raeke et al., 2016; Johnson et al., 2017; Bercovici et al., 2022). The strong lysis background and the natural DOM background of the seawater samples likely contributed to the difficulties to identify a virus signal out of the dataset. Furthermore, the on-going turnover of especially labile N- and P-rich material in natural seawater by active microbial communities may keep the concentrations of virus compounds under a threshold to be detectable (Hach et al., 2020; Zheng et al., 2021). The preparation of a pure virus-extract free from lysis remnants that meets the criteria for DOM analysis would allow a deeper insight into the question if a DOM signature from a single prophage or viruses from a complex microbial community can be detected at all within the analytical window. By using more targeted approaches or by adjusting sample preparation and routine settings it may be possible to overcome the above-described analytical restrictions, allowing for a detection of a virus signal within the DOM of natural marine samples.

## Data Availability Statement

The datasets presented in this study can be found in online repositories. The names of the repository/repositories and accession number(s) can be found below: Genbank JF974307.1.

## Author Contributions

MH, BH, JN, and BE conceived the study. All authors provided scientific input and suggestions. MH, BH, and BA-G conducted all experiments and analyzed the samples. MS greatly contributed to the scope of the study and gave rigorous feedback to the DOM analyses. MH, BH, and BE drafted manuscript with input from all co-authors. All authors were involved in revision and approval of the final version of the manuscript.

## Conflict of Interest

The authors declare that the research was conducted in the absence of any commercial or financial relationships that could be construed as a potential conflict of interest.

## Publisher’s Note

All claims expressed in this article are solely those of the authors and do not necessarily represent those of their affiliated organizations, or those of the publisher, the editors and the reviewers. Any product that may be evaluated in this article, or claim that may be made by its manufacturer, is not guaranteed or endorsed by the publisher.
