# Supplementary material for: Deciphering the Virus Signal Within the Marine Dissolved Organic Matter Pool
Source: Front Microbiol. 2022 May 27;13:863686. doi: 10.3389/fmicb.2022.863686 (PMC9184803; doi:10.3389/fmicb.2022.863686)
Supplement: Supplementary file 1 [file Data_Sheet_1.pdf]

frontiers

Supplementary material to

Deciphering the virus signal within the marine dissolved organic matter pool

Mara E. Heinrichs[1+], Benedikt Heyerhoff[1+], Berin S. Arslan-Gatz[1], Michael Seidel[2], Jutta Niggemann[2], Bert Engelen[1*]

[1]Benthic Microbiology Group, Institute for Chemistry and Biology of the Marine Environment, Carl von Ossietzky University, Oldenburg, Germany

[2]Research Group for Marine Geochemistry (ICBM-MPI Bridging Group), Institute for Chemistry and Biology of the Marine Environment, Carl von Ossietzky University, Oldenburg, Germany

16S rRNA sequence of *R. sulphidophilum*

TATCGGGTAGAACCAACTCCCATGGTGTGACGGGCGGTGTGTACAAGGCCCGGGAACGTATTCACCGCGTC
ATGCTGTTACGCGATTACTAGCGATTCCGACTTCATGGGGTCGAGTTGCAGACCCCAATCCGAACTGAGAC
AGCTTTTTGGGATTATCCCATTGTCACTGCCATTGTAGCACGTGTGTAGCCCAACCCGTAAGGGCCATGAGG
ACTTGACGTCATCCACACCTTCCTCCGACTTATCATCGGCAGTTTCCCTAGAGTGCCCAACTGAATGATGGC
AACTAAGGACGTGGGTTGCGCTCGTTGCCGGACTTAACCGAACATCTCACGACACGAGCTGACGACAGCCA
TGCAGCACCTGTCACTGATCCAGCCGAACTGAAGGAAACCATCTCTGGTAACCGCGATCAGGATGTCAAGG
GTTGGTAAGGTTCTGCGCGTTGCTTCGAATTAAACCACATGCTCCACCGCTTGTGCGGGCCCCCGTCAATTC
CTTTGAGTTTTAATCTTGCGACCGTACTCCCCAGGCGGAATGCTTAATCCGTTAGGTGTGTCACCGAATAGC
ATGCTACCCGACGACTGGCATTCATCGTTTACGGCGTGGACTACCAGGGTATCTAATCCTGTTTGCTCCCCA
CGCTTTCGCACCTCAGCGTCAGTATCGAGCCAGTGAGCCGCCTTCGCCACTGGTGTTCCTCCGAATATCTAC
GAATTTCACCTCTACACTCGGAATTCCACTCACCTCTCTCGAACTCAAGACTACCAGTATCAGAGGCAGTTC
CGGGGTTGAGCCCCGGGATTTCACCCCTGACTTAATAGTCCGCCTACGTGCGCTTTACGCCCAGTAATTCCG
AACAACGCTAGCCCCCTCCGTATTACCGCGGCTGCT

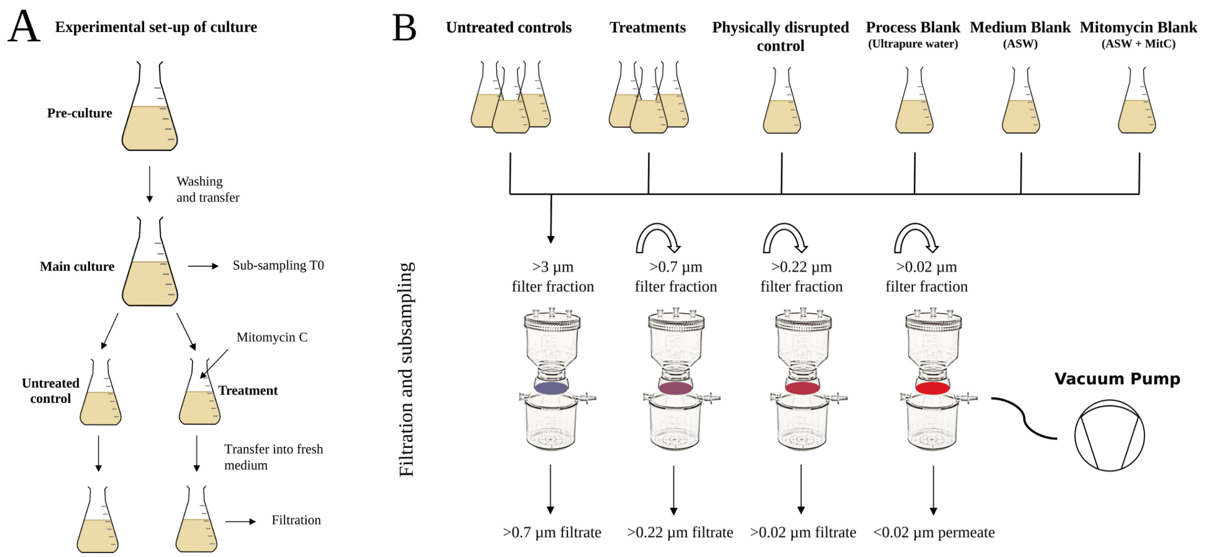

Supplementary Figure S1. Experimental setup of the *R. sulphidophilum* culture. Schematic overview of the individual steps from growing the pre-culture to the filtration (A). The right panel shows all treatments, controls, analytical blanks and filtration steps (B). Prophages of *R. sulphidophilum* cultures were induced via mitomycin C treatment ('treatments') and compared to untreated control cultures. The physically disrupted control was subjected to freeze-and-thaw to lyse the bacterial cells. Ultrapure water ('process blank'), artificial seawater (ASW) with and without mitomycin C (MitC) amendment served as blanks for the DOM analysis. All setups were serial filtered through filters of 3 µm, 0.7 µm, 0.22 µm and 0.02 µm pore sizes.

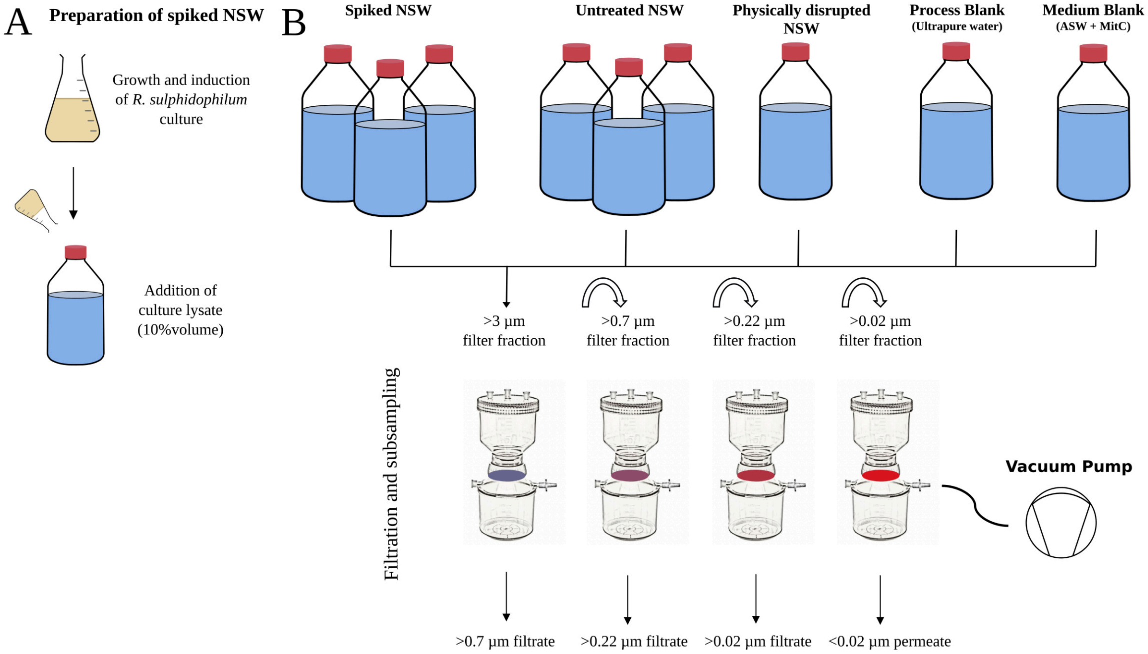

Supplementary Figure S2. Schematic overview of the preparation of the spiked NSW incubations (A) and experimental setup of the North Sea water (NSW) incubations, including all treatments, controls, blanks and filtration steps. The dissolved organic matter composition of natural NSW ('untreated NSW') was compared to NSW spiked with a prophage-induced *R. sulphidophilum* culture. The physically disrupted control was subjected to freeze-and-thaw to lyse the bacterial cells. Ultrapure water ('process blank') and artificial seawater (ASW) amended with mitomycin C (MitC) ('medium blank') served as analytical blanks. All setups were serial filtered through filters of 3 µm, 0.7 µm, 0.22 µm and 0.02 µm pore sizes.

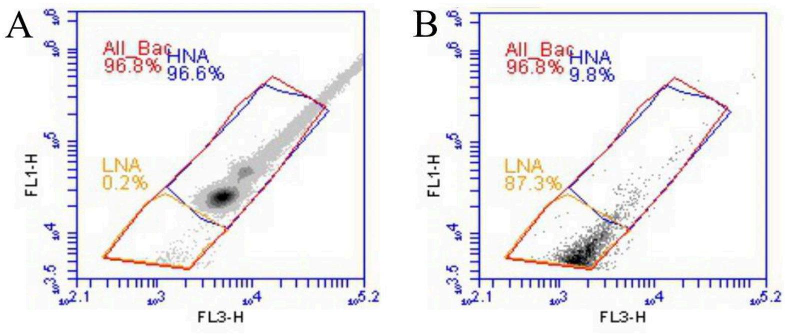

Supplementary Figure S3. Representative cytograms of SYBR Green I stained bacterial cells of the *R. sulphidophilum* culture. Cytograms exported from the BD Accuri C6 software show high nucleic acid (HNA) and low nucleic acid (LNA) subpopulations of the control (A) and the corresponding mitomycin C treated 0.22 µm filter fractions (B). Displayed is the green fluorescence (FL1, 530±15 nm) vs. red fluorescence (FL3, >670 nm).

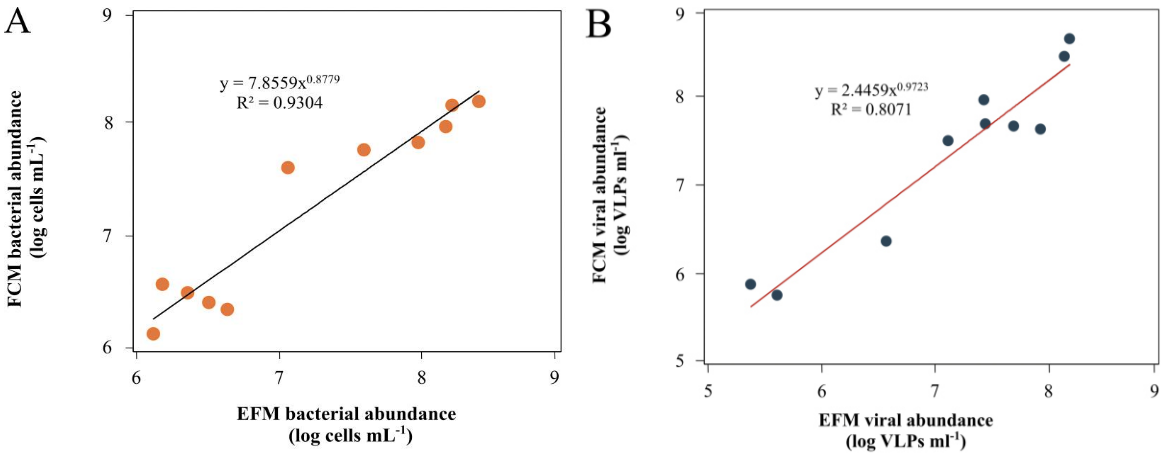

Supplementary Figure S4. Linear regression of bacteria (A) and virus numbers (B), determined by epifluorescence microscopy (EFM) and flow cytometry (FCM).

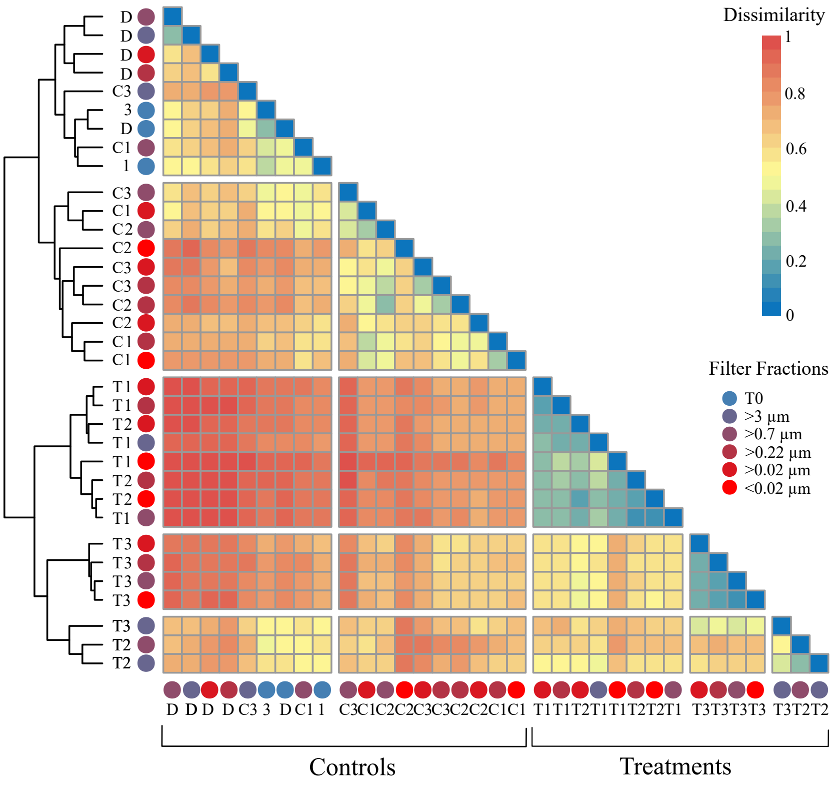

Supplementary Figure S5. Bray-Curtis dissimilarity matrix based on the relative peak intensities of all molecular formulae detected in each sample of the culture setup. The color scale of the heatmap displays the dissimilarity between the samples, and the color scale of the circles the pore size of the filter fractions. Shown are all filter fractions of the treatment (T1-3), the corresponding untreated controls (C1-3) and the disruption controls (D).

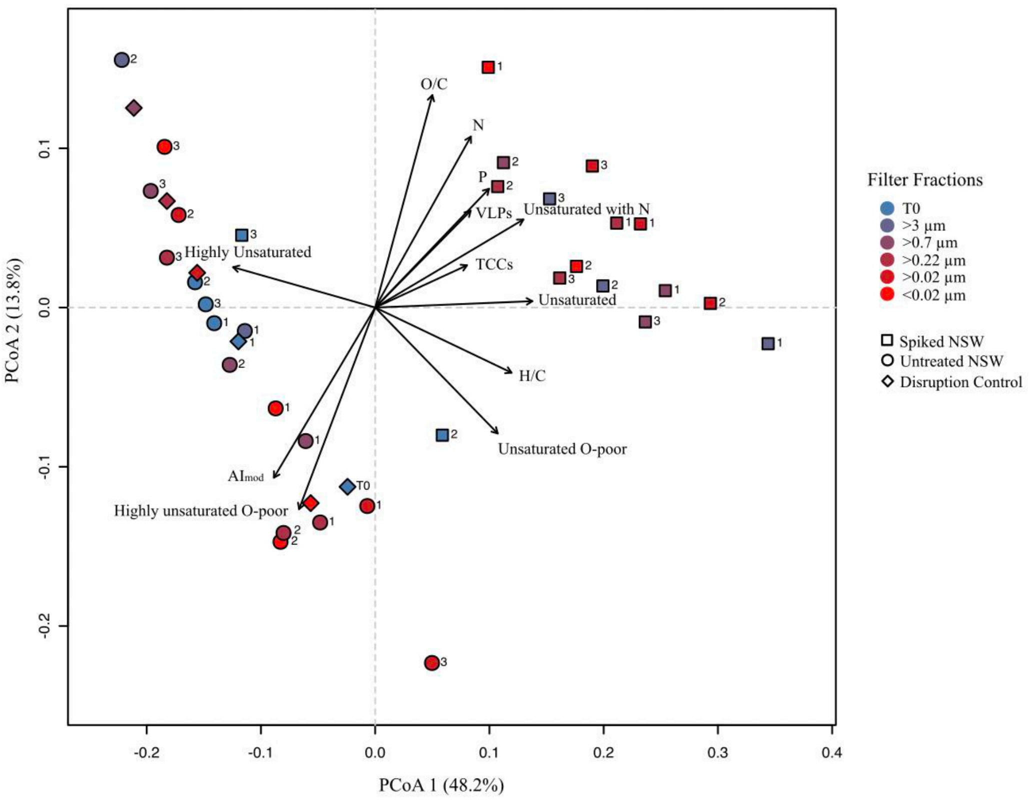

Supplementary Figure S6. Principal coordinate analysis of the dissolved organic matter composition of the North Sea water (NSW) experiment. Circles indicate natural NSW (untreated NSW), squares indicate NSW spiked with cell lysate from a bacterial culture of *R. sulphidophilum* and diamond shapes the disruption control. All samples were sequentially filtered over filters with different pore sizes as indicated by the color code. Black arrows display chemical or experimental variables that significantly ($p < 0.0001$) correlate with principal coordinates (VLPs: virus-like particles, TCCs: cell numbers). Biological replicates are indicated by the numeration 1-3. Displayed are the first two major axes of variation.

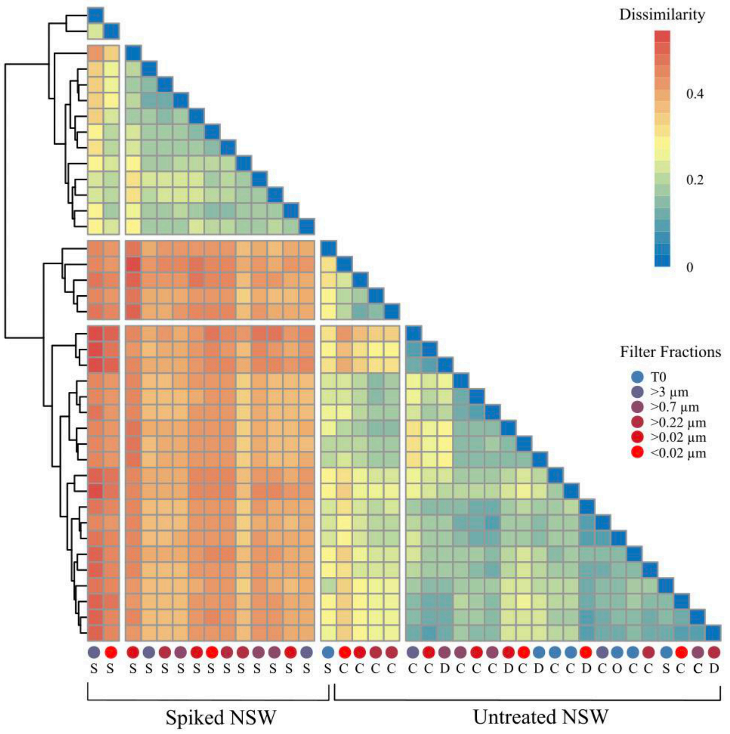

Supplementary Figure S7. Bray Curtis dissimilarity analysis of the North Sea water experiment. The color scale of the heatmap displays the dissimilarity between the samples, and the color scale of the circles the pore size of the filter fractions. Shown are all filter fractions of the natural North Sea water (NSW) (C), NSW spiked with cell lysate from a *R. sulphidophilum* bacterial culture and the disruption controls (D).

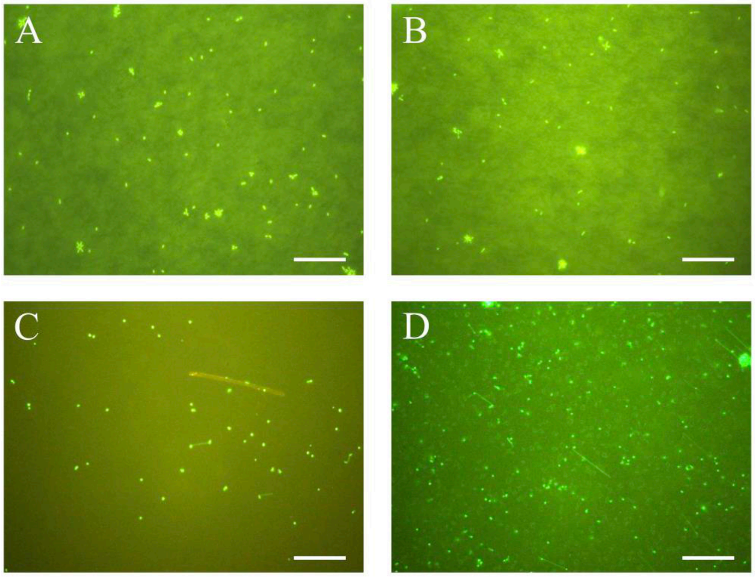

Supplementary Figure S8. Epifluorescence micrographs of *R. sulphidophilum* cells from the untreated control culture (T0, before filtration, A) and mitomycin C treated culture (>3 µm filter fraction, B). Fluorescence images of viruses from the untreated *R. sulphidophilum* culture (C) and corresponding prophage induced culture (D) using mitomycin C. Shown are the >0.02 µm filter fractions. Scale bar represents 200 µm.
